# Supplementary material for: Extended-spectrum β-lactamase and carbapenemase-producing Enterobacterales among adult patients and their family members at Tikur Anbessa Specialized Hospital, Addis Ababa, Ethiopia
Source: PLoS One. 2026 Jan 23;21(1):e0341636. doi: 10.1371/journal.pone.0341636 (PMC12829779; doi:10.1371/journal.pone.0341636)
Supplement: S1 Table — (DOCX) [file pone.0341636.s001.docx]

S1Table

| Multiplex genes | Target genes | Primers | Primer sequence (5’-3’) | Amplicon size |
| --- | --- | --- | --- | --- |
| Group-1 | *bla*_TEM_ | TEM-F | TCAACATTTTCGTGTCGCCC | 766 |
|  |  | TEM-R | AACTACGATACGGGAGGGCT |  |
|  | *bla*_OXA-23-like_ | OXA-F | AGATCCTTGACCCGCAGTTG | 928 |
|  |  | OXA-R | CGCCGTCCCATCGAAAAATC |  |
|  | *bla*_CTX-M_ | CTX-M-F | AGACTGGGTGTGGCATTGAT | 676 |
|  |  | CTX-M-R | TTAGGTTGAGGCTGGGTGAAGT |  |
| Group-2 | *bla*_GES_ | GES-F | TCACTCTGCATATGCGTCGG | 692 |
|  |  | GES-R | ACTTGACCGACAGAGGCAAC |  |
|  | *bla*_VEB_ | VEB-F | CCCGATGCAAAGCGTTATGA | 184 |
|  |  | VEB-R | ACCCCAACATCATTAGTGGC |  |
|  | *bla*_AmpC_ | AmpC-F | GATGAAGGCCAATGACATTCCG | 576 |
|  |  | AmpC-R | CATGTCGCCGACCTTGTAGTAA |  |
|  | *bla*_SHV_ | SHV-F | CGCCTGTGTATTATCTCCCT | 293 [27] |
|  |  | SHV-R | CGAGTAGTCCACCAGATCCT |  |

F= Forward primer

R= Reverse primer
