# Supplementary material for: Extended-spectrum β-lactamase and carbapenemase-producing Enterobacterales among adult patients and their family members at Tikur Anbessa Specialized Hospital, Addis Ababa, Ethiopia
Source: PLoS One. 2026 Jan 23;21(1):e0341636. doi: 10.1371/journal.pone.0341636 (PMC12829779; doi:10.1371/journal.pone.0341636)
Supplement: S2 Table — (DOCX) [file pone.0341636.s002.docx]

S2 Table

| Multiplex genes | Target gen | Primer | Primer sequence (5’-3’) | Amplicon size |
| --- | --- | --- | --- | --- |
| Carba-group-1 | *bla*_KPC_ | KPC-Fm | CGTCTAGTTCTGCTGTCTTG | 798 |
|  |  | KPC-Rm | CTTGTCATCCTTGTTAGGCG | 232 |
|  | *bla*_NDM_ | NDM-F | GGTTTGGCGATCTGGTTTTC | 621 |
|  |  | NDM-R | CGGAATGGCTCATCACGATC |  |
|  | *bla*_OXA-48_ | OXA-F | GCGTGGTTAAGGATGAACAC | 438 |
|  |  | OXA-R | CATCAAGTTCAACCCAACCG |  |
|  | *bla*_BIC_ | BIC-F | TATGCAGCTCCTTTAAGGGC | 537 |
|  |  | BIC-R | TCATTGGCGGTGCCGTACAC |  |
| Carba-group-2 | *bla*_IMP_ | IMP-F | GGAATAGAGTGGCTTAAYTCTC | 232 |
|  |  | IMP-R | GGTTTAAYAAAACAACCACC |  |
|  | *bla*_SPM_ | SPM-F | AAAATCTGGGTACGCAAACG | 271 |
|  |  | SPM-R | ACATTATCCGCTGGAACAGG |  |
|  | *bla*_VIM_ | VIM-F | GATGGTGTTTGGTCGCATA | 390 |
|  |  | VIM-R | CGAATGCGCAGCACCAG |  |
| Carba-group-3 | *bla*_AIM_ | AIM-F | CTGAAGGTGTACGGAAACAC | 322 |
|  |  | AIM-R | GTTCGGCCACCTCGAATTG |  |
|  | *bla*_GIM_ | GIM-F | TCGACACACCTTGGTCTGAA | 477 |
|  |  | GIM-R | AACTTCCAACTTTGCCATGC |  |
|  | *bla*_SIM_ | SIM-F | TACAAGGGATTCGGCATCG | 570 |
|  |  | SIM-R | TAATGGCCTGTTCCCATGTG |  |
|  | *bla*_DIM_ | DIM-F | GCTTGTCTTCGCTTGCTAACG | 699 |
|  |  | DIM-R | CGTTCGGCTGGATTGATTTG |  |

F= Forward primer

R= Reverse primer
