# Supplementary material for: Extended-spectrum β-lactamase and carbapenemase-producing Enterobacterales among adult patients and their family members at Tikur Anbessa Specialized Hospital, Addis Ababa, Ethiopia
Source: PLoS One. 2026 Jan 23;21(1):e0341636. doi: 10.1371/journal.pone.0341636 (PMC12829779; doi:10.1371/journal.pone.0341636)
Supplement: Attachment — Submitted filename: Comments.docx [file pone.0341636.s003.docx]

First I would like to thank PLOS One editors for inviting me to review this paper. I have comments for consideration during decision.

**Title:**

Β-lactamase producing Enterobacterales among adult patients and their family members at Tikur Anbessa Specialized Hospital, Addis Ababa, Ethiopia

- The title is interesting because it include traced study participants with index patients. And this could generate important evidence.

**Abstract**

Well written and address all expectation in this section. However, there is redundancy in the result and conclusion, please review.

**Introduction**

The introduction is good. However, the rational of the study need more clarification. There are similar study done in the community. Please indicate the gaps you want to fill in this regard. The justification look the same for ESBL-PE and CRE; in the title only indicated ESBL-PE, why not CRE.

**Methods and Materials**

In the method section, how you determine the sample size is not indicated. Somewhere in document sample size was 100, how you come up with this number? In the bacterial identification you mentioned using conventional method and VITEK 2 Compact system (bioMe´rieux, France). How you interprete the result is not explained.

In the PCR assay, you applied multiplex PCR assay. How you grouped the primers of the interest, based on what?

**Results**

Well presented
