# Supplementary material for: Extended-spectrum β-lactamase and carbapenemase-producing Enterobacterales among adult patients and their family members at Tikur Anbessa Specialized Hospital, Addis Ababa, Ethiopia
Source: PLoS One. 2026 Jan 23;21(1):e0341636. doi: 10.1371/journal.pone.0341636 (PMC12829779; doi:10.1371/journal.pone.0341636)
Supplement: Attachment — Submitted filename: Review Comments to the Author PONE-D-25-40715.docx [file pone.0341636.s004.docx]

**Review Comments to the Author**

**PONE-D-25-40715**

β-lactamase producing Enterobacterales among adult patients and their family members at Tikur Anbessa Specialized Hospital, Addis Ababa, Ethiopia

**Dear Editor,**

Thank you for inviting me to review this manuscript on β-lactamase-producing Enterobacterales among adult patients and their family members at Tikur Anbessa Specialized Hospital, Addis Ababa, Ethiopia.

General comments

1. The paper has typographical errors. It should be corrected.
2. Clarification needed for sample size calculation.
3. Some genes classified as ESBL, namely blaOXA, blaVIM, and blaDIM, are actually carbapenemase genes, while blaAmpC belongs to the AmpC β-lactamase category and is distinct from ESBLs.
4. Tables showing phenotyping results along with chi-square, bivariate, and multivariate p-values should be included in the manuscript.
5. The study site, Tikur Anbessa Specialized Hospital, should be properly acknowledged.

Specific comments

1. Title β-lactamase-producing Enterobacterales among adult patients and their family members at Tikur Anbessa Specialized Hospital, Addis Ababa, Ethiopia
   - It seems only betalactamse. Where is carbapenemase production in the title
2. Abstract part:
   - Better to change “extended spectrum β-lactamases to Extended**-**spectrum β-Lactamases and carbapenem resistant Enterobacterales (CRE) to Carbapenem**-**resistant Enterobacterales (CRE) throughout the document. If you once used in bracket in the Abstract and introduction, the rest should be the abbreviation. You are not supposed to repeat the full and the abbreviation in brackets in the document. Line 169, 404
   - Of the ESBL-PE isolates, 84.6% from patients and 100% from family members carried at least one ESBL encoding gene, with *bla*CTX-M being the predominant. How can it be that ESBL-PE isolates are greater in family members than in patients? Taking treatment for patients may vary in results. (Line 36-38)
   - Do not use abbreviations at the first appearance (CP-CRE), line 39 change being to are, in (line 40).
   - Use a similar pattern for (P:0.02), (P= 0.028), (P<0.001). better to use (P = 0.028) or p-value and (P < 0.001) throughout the document. (Lines 35, 40, 41, 309, and 334 in the result part).
3. Introduction typographical error
   - line 56, correct “carbapenem resistant- carbapenemase producing Enterobacterales (**CP-CRE**) ” to “**Carbapenemase-Producing Carbapenem-Resistant Enterobacterales (CP-CRE)”**
   - Line 64. Change sever infections to sever**e** infections
   - Line 65. Do not start a sentence with an abbreviation
   - Line 66. Change carbapenmase encoding to carbapen**e**mase encoding
   - Line 76. Avoid a full stop after the word while**.**
   - Line 60, 77, 83, 87, 93, 117, 205, 211, 233, and 356 insert a comma before the word and.
   - Line 119 “change lysine mannitol, triple sugar iron (TSI)” to lysine**,** mannitol, **and** triple sugar iron (TSI).
4. Materials and methods
   - What is your sample size calculation? Is it a representative sample?
   - Why do you do the manual and Vitek 2 biochemical tests twice?
   - Line 146 correct loop to loops
   - Line 147, remove a full stop before and.
   - Line 152-155 correct “Measure of inhibition zone with diameter of 6–15 mm or pinpoint colonies were determined as positive for carbapenemase enzyme production; and a zone of inhibition ≥19 mm was considered to be negative for carbapenemase enzyme production.” Change to “Measure of inhibition zone with diameter of 6–15 mm or pinpoint colonies in **16–18 mm** were determined as positive for carbapenemase enzyme production; and a zone of inhibition ≥19 mm was considered to be negative for carbapenemase enzyme production.”
   - Use a reference for phenotypic detection of ESBL production in Line 138.
   - Use a reference for **Bacterial DNA extraction;** otherwise, put your validation result Line 158.
   - Line 171 Change *bla*AmPC to *bla* AmpC
   - Line 187 table 1 ESBL category: blaTEM, blaOXA, blaCTX-M, blaVIM, blaGES, blaVEB, blaDIM, blaAmpC, blaSHV
   - In lines 170 and 187, how are blaOXA, blaDIM, and blaVIM classified as ESBLs when they are not ESBL genes?
     - The blaOXA, blaVIM, and blaDIM genes encode carbapenemase enzymes that mediate resistance to carbapenems. Specifically, blaVIM and blaDIM encode metallo-β-lactamases (MBLs) that require zinc ions and can hydrolyze carbapenems along with a broad range of β-lactams.
     - The blaOXA primer pair (Forward: AGATCCTTGACCCGCAGTTG; Reverse: CGCCGTCCCATCGAAAAATC) targets blaOXA-23–like genes, commonly found in Acinetobacter species. OXA-type enzymes, including blaOXA-23, blaOXA-24/40, blaOXA-51, and blaOXA-58, are oxacillinases capable of hydrolyzing carbapenems and other β-lactams, contributing to carbapenem resistance.
     - Why is AmpC categorized under ESBL? AmpC β-lactamase–producing organisms typically show resistance to second- and third-generation cephalosporins (e.g., cefuroxime, cefotaxime, ceftazidime) as well as β-lactam/β-lactamase inhibitor combinations, which differs from ESBL classification.
   - It would be better to add *blaPER* and *blaBEL* ESBL genes
   - In line 196, Table 2 lists of the carbapenemase genes blaKPC, blaNDM, blaOXA-48, blaBIC, blaIMP, blaSPM, blaVIM, blaAIM, blaGIM, blaSIM, and blaDIM.
   - Why is blaDIM included under both ESBL and carbapenemase categories? It would be more appropriate to remove it from the ESBL list.
5. Results
   - Correct Line 282, the word Hemorriegic to Hemorrhagic
   - Insert the phynotyping result of the chi-square, bivariate analysis, and multivariate analysis tables.
6. Reference
   - References 21, 22, 26, and 28 are older sources; however, they are acceptable since they describe the **original method.**
