# Supplementary material for: Extended-spectrum β-lactamase and carbapenemase-producing Enterobacterales among adult patients and their family members at Tikur Anbessa Specialized Hospital, Addis Ababa, Ethiopia
Source: PLoS One. 2026 Jan 23;21(1):e0341636. doi: 10.1371/journal.pone.0341636 (PMC12829779; doi:10.1371/journal.pone.0341636)
Supplement: Attachment — Submitted filename: Response to reviewer-2.docx [file pone.0341636.s005.docx]

**Response to reviewers**

**Journal: *PLoS ONE***

**ID; PONE-D-25-40715**

Title: Extended-spectrum β-Lactamase and Carbapenemase-producing *Enterobacterales* among adult patients and their family members at Tikur Anbessa Specialized Hospital, Addis Ababa, Ethiopia

**We sincerely thank the reviewers and editors for their thorough evaluation of our manuscript and providing valuable comments and suggestions, which would improve the paper's quality in all directions. We have carefully addressed all the comments and suggestions given by reviewers and editors. For clarity, all changes or revisions have been highlighted in blue with in the document. Furthermore, all authors have reviewed the revised version to ensure that the comments and suggestions were fully incorporated. We believe these revisions have improved the quality, readability, and clarity of the manuscript.**

**Review Comments to the Author**

**PONE-D-25-40715**

β-lactamase producing Enterobacterales among adult patients and their family members at Tikur Anbessa Specialized Hospital, Addis Ababa, Ethiopia

**Dear Editor,**

Thank you for inviting me to review this manuscript on β-lactamase-producing Enterobacterales among adult patients and their family members at Tikur Anbessa Specialized Hospital, Addis Ababa, Ethiopia.

General comments

1. The paper has typographical errors. It should be corrected.
2. Clarification needed for sample size calculation.
3. Some genes classified as ESBL, namely blaOXA, blaVIM, and blaDIM, are actually carbapenemase genes, while blaAmpC belongs to the AmpC β-lactamase category and is distinct from ESBLs.
4. Tables showing phenotyping results along with chi-square, bivariate, and multivariate p-values should be included in the manuscript.
5. The study site, Tikur Anbessa Specialized Hospital, should be properly acknowledged.

Specific comments

1. Title β-lactamase-producing Enterobacterales among adult patients and their family members at Tikur Anbessa Specialized Hospital, Addis Ababa, Ethiopia
   - It seems only betalactamse. Where is carbapenemase production in the title

Thank you for your insightful comment. β-lactamase is a broad term that can include carbapenemases, however we agree that this may not clearly reflect the specific focus on carbapenemase. Therefore, we have revised our title to indicate carbapeneamse producing Enterobacterales as outlined below (page 1, line 1-2):

Extended-spectrum β-Lactamases and Carbapenemase-producing *Enterobacterales* among adult patients and their family members at Tikur Anbessa Specialized Hospital, Addis Ababa, Ethiopia

2. Abstract part:
   - Better to change “extended spectrum β-lactamases to Extended**-**spectrum β-Lactamases and carbapenem resistant Enterobacterales (CRE) to Carbapenem**-**resistant Enterobacterales (CRE) throughout the document. If you once used in bracket in the Abstract and introduction, the rest should be the abbreviation. You are not supposed to repeat the full and the abbreviation in brackets in the document. Line 169, 404

Thank you. We have corrected it based on your recommendation as indicted in the abstract section below (page 2, line 25-27), and throughtout the document:

Extended-spectrum-β-Lactamases-producing Enterobacterales (ESBL-PE), carbapenem-resistant Enterobacterales (CRE).

We also changed the full term of Extended-spectrum β-Lactamaseses (ESBLs) into β-Lactamase genes in page 10, line 191), and ESBLs in page 32, line 410), and through out the document

- Of the ESBL-PE isolates, 84.6% from patients and 100% from family members carried at least one ESBL encoding gene, with *bla*CTX-M being the predominant. How can it be that ESBL-PE isolates are greater in family members than in patients? Taking treatment for patients may vary in results. (Line 36-38)

Thank you so much for your comment. Antibiotic treatment in patients may suppress (<https://doi.org/10.1186/s12866-020-01741-7>), or enhance resistance gene detectionhttps (https://doi.org/10.3390/ antibiotics11070953). Therefore, antibiotic treatment may affect our gene detection among patients, while family members were not on antibioitcs, hence 100% gene detection. The other reason may be related with low number of ESBL-PE isolates in family members, which were 24 ESBL-PE isolates compared to 39 ESBL-PE isolates among patients.

- Do not use abbreviations at the first appearance (CP-CRE), line 39 change being to are, in (line 40).

Thank you. We have corrected it as indicated below in the abstract section (page 2, line 39-42):

Colonization with CRE and Carbapenemase-Producing Carbapenem-Resistant *Enterobacterales* was found to be 19.0% and 10.0%, respectively. These were identified only among patients, with *bla*_NDM_ and *bla*_OXA-48_ are the most prevalent genes.

- Use a similar pattern for (P:0.02), (P= 0.028), (P<0.001). better to use (P = 0.028) or p-value and (P < 0.001) throughout the document. (Lines 35, 40, 41, 309, and 334 in the result part).

We thank you. We have corrected it as P=0.028), (P=0.02) and (P<0.001) through out the document and highlited with blue color.

3. Introduction typographical error
   - line 56, correct “carbapenem resistant- carbapenemase producing Enterobacterales (**CP-CRE**) ” to “**Carbapenemase-Producing Carbapenem-Resistant Enterobacterales (CP-CRE)”**

**We thank you. We have corrected it as:** **“**Carbapenemase-Producing Carbapenem-Resistant Enterobacterales (CP-CRE)”**, indicated in the introduction section (page 4, line 56-57), as indicated below:**

Extended-spectrum-β-lactamases (ESBLs) and Carbapenemase-Producing Carbapenem-Resistant *Enterobacterales* (CP-CRE).

- Line 64. Change sever infections to sever**e** infections

Thank you. We have modified it as “severe” as indictaed in the introduction section (page 4, line 64), and outlined below:

Carbapenems are often taken as the last resort antibiotics for severe infections caused by multi-drug resistant (MDR) pathogens.

- Line 65. Do not start a sentence with an abbreviation

We thank you very much. We have modified it as **“**Carbapenemase-Producing Carbapenem-Resistant Enterobacterales (CP-CRE)” ( page 4, line 65-66)

- Line 66. Change carbapenmase encoding to carbapen**e**mase encoding

Thank you. We have corrected it as carbapenemase encoding genes (page 4, line 67)

- Line 76. Avoid a full stop after the word while

**Thank you. We removed it (page 5, line 77)**

- Line 60, 77, 83, 87, 93, 117, 205, 211, 233, and 356 insert a comma before the word and.

Thank you for these corrections. We have insereted comma before “and” in the introduction section (line 61, 78, 84, 89). In the materials and methodology section (line 94, 138, 213, 219, 238). In the result section (line 353).

- Line 119 “change lysine mannitol, triple sugar iron (TSI)” to lysine**,** mannitol, **and** triple sugar iron (TSI).

We appreciate. We have modified this section based on your suggestion as indicated below ( page 8, line 140), as indicated below:

Then a single pure colony was sub-cultured on tryptic soya agar (TSA), and the identity of the target bacteria was confirmed by biochemical tests such as indole production, urease, oxidase, citrate, lysine, mannitol, and triple sugar iron (TSI).

4. Materials and methods
   - What is your sample size calculation? Is it a representative sample?

Thank you for your insight full comment. We acknowledge that the sample size determination and sampling technique were unintentionally omitted from the submitted version. We have now included in the revised version by calculating the sample size using a case-control formula based on previous prevalence of ESBL colonization among hospitalized patients and healthy volunteers, and by considering 80% power, and 95% confidence interval, which ensures that the study has adequeate statistical power to detect meaningful differences between patients and their family members. Additionally, we used systematic random sampling technique, which ensures that every eligible participant has equal chance of being selected (page 6-7, line 102-121), as outlined below:

The sample size was calculated using case-control calculation formula, n= (r+1) ×p(1-p) × (Zα/2+Zβ)^2^ /r (P1-P2)^2,^ by considering the previous prevalence of ESBL-PE colonization among hospitalized patients (P1=42%) and healthy volunteers (P2=22%) in Burkina Faso. The value of 𝑍𝛼/2 is 1.96 at 95% confidence level, and Zβ is set at the critical value of 0.84 with a margin of 5%. By including the 20% non-response rate, the final sample size was determined to be 98 for each participant, which gave a total of 204 sample size.

P1= Prevalence of ESBL colonization among hospitalized patients

P2=Prevalence of EBL colonization among healthy volunteers

P= Average prevalence (P1+P2)/2

r= Ratio of control to cases (1 for equal number of control and cases)

P1-P2= Effect size or different in proportion expected based on previous study

A systematic random sampling technique was used to select study participants aged 18 years and above who were admitted to internal medicine wards. We used the list of all patients who were admitted to medical wards over the past six months for the purpose of using it as our sampling frame. The total number (N) of admitted patients in the past six months was 624, and the minimum calculated sample size (n) was **204**. Therefore, K= N/n: 624/204=3. A random start was chosen from the first three admissions, and subsequently every third patient was enrolled until the desired sample size was achieved. However, four eligible study participants (both patients and family members) declined to participate. Therefore, a total of 200 stool samples were collected.

- Why do you do the manual and Vitek 2 biochemical tests twice?

Thank you so much for this comment. We used both manual and VITEK 2 biochemical tests to cross-check any discrepancies, and to strengthen the overall quality and confidence of our identification process, and final results were reported based on Vitek 2 system.

- Line 146 correct loop to loops

Thank you we have changed loop to “loops” ( Page 9, line 168)

- Line 147, remove a full stop before and.

Thank you. We removed it (page 9, line 169)

- Line 152-155 correct “Measure of inhibition zone with diameter of 6–15 mm or pinpoint colonies were determined as positive for carbapenemase enzyme production; and a zone of inhibition ≥19 mm was considered to be negative for carbapenemase enzyme production.” Change to “Measure of inhibition zone with diameter of 6–15 mm or pinpoint colonies in **16–18 mm** were determined as positive for carbapenemase enzyme production; and a zone of inhibition ≥19 mm was considered to be negative for carbapenemase enzyme production.”

Thank you for this comment. We have modified this section based on your suggestion ( page 9, line 174-177), as indicated below:

Measure of inhibition zone with diameter of 6–15 mm or pinpoint colonies in 16-18 mm were determined as positive for carbapenemase enzyme production; and a zone of inhibition ≥19 mm was considered to be negative for carbapenemase enzyme production.

- Use a reference for phenotypic detection of ESBL production in Line 138.

Thank you. We have included the reference for this section in ( page 9, line 165). Pincus DH. Microbial identification using the bioMérieux Vitek® 2 system. Encyclopedia of Rapid Microbiological Methods Bethesda, MD: Parenteral Drug Association2006. p. 1-32.

- Use a reference for **Bacterial DNA extraction;** otherwise, put your validation result Line 158.

Thank you. We have added the reference on this section (page 10, line 181).

Kpoda DS, Ajayi A, Somda M, Traore O, Guessennd N, Ouattara AS, et al. Distribution of resistance genes encoding ESBLs in Enterobacteriaceae isolated from biological samples in health centers in Ouagadougou, Burkina Faso. BMC Res Notes

- Line 171 Change *bla*AmPC to *bla*AmpC

Thank you. We changed *bla*_AmPC_ to *bla*_AmpC_ ( Line 192).

- Line 187 table 1 ESBL category: blaTEM, blaOXA, blaCTX-M, blaVIM, blaGES, blaVEB, blaDIM, blaAmpC, blaSHV
- In lines 170 and 187, how are blaOXA, blaDIM, and blaVIM classified as ESBLs when they are not ESBL genes? .
  - The blaOXA, blaVIM, and blaDIM genes encode carbapenemase enzymes that mediate resistance to carbapenems. Specifically, blaVIM and blaDIM encode metallo-β-lactamases (MBLs) that require zinc ions and can hydrolyze carbapenems along with a broad range of β-lactams.

Thank you pointing out this important issue. We agree that blaOXA, blaVIM, blaDIM, and blaAmpC, genes are not ESBL genes. β-Lactamase is a abroad term that includes both ESBL and carbapenemase encoding genes. Therfore, we revised this table title as “Primer sequences used for amplification of β-Lactamase genes” (S1 Table), and “Primer sequences used for amplification of CP-CRE genes (page 11, line 209-210). We adopt this protocols from previous studies that target broad range of β-lactamase genes, including ESBLs, AmpC, and carbapenemases (doi: 10.1016/j.kjms.2017.09.010. Epub 2017 Nov 6); and carbapenemase genes (doi: 10.1016/j.diagmicrobio.2010.12.002).

- The blaOXA primer pair (Forward: AGATCCTTGACCCGCAGTTG; Reverse: CGCCGTCCCATCGAAAAATC) targets blaOXA-23–like genes, commonly found in Acinetobacter species. OXA-type enzymes, including blaOXA-23, blaOXA-24/40, blaOXA-51, and blaOXA-58, are oxacillinases capable of hydrolyzing carbapenems and other β-lactams, contributing to carbapenem resistance.

Thank you very much for this insightful comment. Yes you are correct, this primer sequence is used specifically targeted blaOXA-23-like genes. Therefore, we replaced blaOXA with blaOXA-23-like genes ( S1 Table, and page 11, line 210).

- Why is AmpC categorized under ESBL? AmpC β-lactamase–producing organisms typically show resistance to second- and third-generation cephalosporins (e.g., cefuroxime, cefotaxime, ceftazidime) as well as β-lactam/β-lactamase inhibitor combinations, which differs from ESBL classification.

We appreciate for your comment. Yes, you are right, AmpC β-lactamase is not under ESBL classification. Thetefore, we renamed the table as β-Lactamase genes that includes the AmpC β-lactamase as indicated in (S1 Table and page 11, line 209-210).

- It would be better to add *blaPER* and *blaBEL* ESBL genes

Thank you for your valuable suggestion. However, the multiplex PCR assay used in our study was adopted from previous study, which did not include primers for blaPER and blaBEL ESBL genes. Therfore, we preferred the title to be “Primer sequences used for amplification of β-Lactamase genes” and “Primer sequences used for amplification of CP-CRE genes” (S1Table, and page 11, line 209-210), and this is also found in the supplementary file (page 47, line 717-719). Therefore, we kindly request you to accept this clarification.

- In line 196, Table 2 lists of the carbapenemase genes blaKPC, blaNDM, blaOXA-48, blaBIC, blaIMP, blaSPM, blaVIM, blaAIM, blaGIM, blaSIM, and blaDIM.
- Why is blaDIM included under both ESBL and carbapenemase categories? It would be more appropriate to remove it from the ESBL list.

Thank you for this comment. We have removed blaDIM from β-Lactamase list in supplimantry file (S1Table). Now it is included in the Carbapenemases encoding genes list (S 2 Table and page 11, line 210).

5. Results
   - Correct Line 282, the word Hemorriegic to Hemorrhagic

Thank you. We have changed it in ( Table 2, line 271)

- Insert the phynotyping result of the chi-square, bivariate analysis, and multivariate analysis tables.

Thank you for this important comment. We have revised our tables by including chi-square, bivariate and multivariate analysis (Table 3, 4, 6, 7 and 8). However, we didn’t include the multivariable analysis in some of our tables baceause of limited number of independent variables, which may potentially mislead readers. Furthermore, anay results with values below 5 were omitted from the statistical analysis.

We have also acknowledged Tikur Anbessa specialized hospital (Acknowledgment section, line 561-562), as indicated below:

We would like to extend our sincere appreciation to Addis Ababa University, Tikur Anbessa specialized hospital, Ethiopian Public Health Institute, Armauer Hansen Research Institute, and Arsho Diagnostic Laboratory for providing the essential materials for this research. Our gratitude also goes to the study participants and data collectors for their willingness to engage in this research endeavor.

6. Reference
   - References 21, 22, 26, and 28 are older sources; however, they are acceptable since they describe the **original method.**

Thank you for pointing this out. We agree that the cited references are older sources, however, they describe the original and standardize methodologies. Specifically, reference 21 is a classical and widely accepted manual, which is available in the EPHI laboratory for manual biochemical identification; and reference 22 provides very detailed description of microbial identification using the Vitek 2 compact system; reference 26 is the protocol that we used during our analysis; refernce 28 is internationally accepted definition for multidrug-resistant, extensively drug-resistant, and pandrug-resistant bacteria. Despite their publication dates the references are appropriate and relevant. Therfore, we kindly request you to accept our justification.
